# Ketamine-mediated afferent-specific presynaptic transmission blocks in low-threshold and sex-specific subpopulation of myelinated Ah-type baroreceptor neurons of rats

**DOI:** 10.18632/oncotarget.6586

**Published:** 2015-12-12

**Authors:** Lu-Qi Wang, Sheng-Zhi Liu, Xin Wen, Di Wu, Lei Yin, Yao Fan, Ye Wang, Wei-Ran Chen, Pei Chen, Yang Liu, Xiao-Long Lu, Hong-Li Sun, Weinian Shou, Guo-Fen Qiao, Bai-Yan Li

**Affiliations:** ^1^ Department of Pharmacology, Harbin Medical University, Harbin, China; ^2^ Key Laboratory of Cardiovascular Medicine Research of Ministry of Education, Harbin Medical University, Harbin, China; ^3^ Department of Pharmacology, Daqing Campus of Harbin Medical University, Daqing, China; ^4^ Riley Heart Research Center, Division of Pediatric Cardiology, Herman B. Wells Center for Pediatric Research, Department of Pediatrics, Indiana University School of Medicine, Indianapolis, IN, USA

**Keywords:** nodose ganglia (NG), nucleus of the solitary tract (NTS), presynaptic neurotransmission, baroreflex afferent pathway, ketamine (Ket), Pathology Section

## Abstract

**Background:**

Ketamine enhances autonomic activity, and unmyelinated C-type baroreceptor afferents are more susceptible to be blocked by ketamine than myelinated A-types. However, the presynaptic transmission block in low-threshold and sex-specific myelinated Ah-type baroreceptor neurons (BRNs) is not elucidated.

**Methods:**

Action potentials (APs) and excitatory post-synaptic currents (EPSCs) were investigated in BRNs/barosensitive neurons identified by conduction velocity (CV), capsaicin-conjugated with Iberiotoxin-sensitivity and fluorescent dye using intact nodose slice and brainstem slice in adult female rats. The expression of mRNA and targeted protein for NMDAR1 was also evaluated.

**Results:**

Ketamine time-dependently blocked afferent CV in Ah-types in nodose slice with significant changes in AP discharge. The concentration-dependent inhibition of ketamine on AP discharge profiles were also assessed and observed using isolated Ah-type BRNs with dramatic reduction in neuroexcitability. In brainstem slice, the 2^nd^-order capsaicin-resistant EPSCs were identified and ∼50% of them were blocked by ketamine concentration-dependently with IC_50_ estimated at 84.4 μM compared with the rest (708.2 μM). Interestingly, the peak, decay time constant, and area under curve of EPSCs were significantly enhanced by 100 nM iberiotoxin in ketamine-more sensitive myelinated NTS neurons (most likely Ah-types), rather than ketamine-less sensitive ones (A-types).

**Conclusions:**

These data have demonstrated, for the first time, that low-threshold and sex-specific myelinated Ah-type BRNs in nodose and Ah-type barosensitive neurons in NTS are more susceptible to ketamine and may play crucial roles in not only mean blood pressure regulation but also buffering dynamic changes in pressure, as well as the ketamine-mediated cardiovascular dysfunction through sexual-dimorphic baroreflex afferent pathway.

## INTRODUCTION

Ketamine has extensively been used in the clinical practice as the anesthetic agents. Increasing evidences have shown that the intravenous anesthetic ketamine increases the blood pressure, heart rate and cardiac output [1-3], alters autonomic nerve activity and baroreflex afferent function [4-7]. Recent study indicates ketamine-mediated the pro-arrhythmic effect and problematic rise in blood pressure [2, 8-10]. Additionally, unusual cases of death caused by acute or chronic ketamine poisoning have been reported [11, 12], and long-term administration of ketamine induces a significant ventricular structural and electrophysiological remodeling [13]. Recently, cardiac arrest following ketamine administration for rapid sequence intubation has been reported in critical ill patients [14], and a significant cardiac and kidney toxicity have also been confirmed by ketamine self-administration in rodents induces cardiotoxicity [15, 16].

Notably, the low-threshold and sex-specific distribution of myelinated Ah-type baroreceptor neurons (BRNs) [17-20] have extensively been studied since the intact nodose slice preparation is developed [21, 22]. As compared with traditionally classified A- and C-types, the afferent conduction and neuroexcitability of this Ah-types are more like A-types, while, the afferent-specific chemosensitivity to vanilloid receptor agonist capsaicin [23] or neurotransmitter histamine [24, 25] are more similar to A-types or C-types, respectively, which may lead at least partially to the sexual-dimorphism in aortic baroreflex function [26]. Even though Ah-types are fast conducted and myelinated afferents, they also share discharge characteristics with unmyelinated C-types, such as, repolarization hump [17], expression tetrodotoxin-resistant Na^+^ channels [18, 27], and large conductive Ca^2+^-activated K^+^ channels [8, 19]. Our previous report have shown that myelinated A-types is less susceptible to ketamine-mediated presynaptic transmission block [4] compared with C-types, however, it would be very interesting to see the distinctive response of this myelinated Ah-type BRNs in nodose ganglia (NG) and the 2^nd^-order Ah-type barosensitive neurons in the nucleus of the solitary tract (NTS) to ketamine due largely to the difference in electrophysiological property and chemosensitivity.

Only one study in published literatures has demonstrated the sex-difference in ketamine-induced cardiac output [3], due mainly to the male species used in the vast majority of previous investigation, so, it stands to reason for lacking of the evidence to directly support the gender difference in ketamine-mediated cardiac physiology and dysfunction. However, several lines of evidence may point out this potential direction: (1) an extensive studies have demonstrated that the low-threshold and sex-specific distribution of myelinated Ah-type BRNs [17-20, 22, 24, 27, 28] services as a functional 1^st^-order neurons in NG and the 2^nd^-order Ah-type barosensitive neurons perhaps in NTS [28]; (2) the neuroexcitability of these Ah-type BRNs, not A- and C-types, is tightly regulated by female hormone and likely responsible for the sexual-dimorphism in baroreflex afferent functions [26]; (3) Ketamine differentially blocks presynaptic sensory afferent neurotransmission [4] in male rats, but no such information is available in female rats so far.

Therefore, this study is designed to test the effects of ketamine on presynaptic neurotransmission in the cell body of the 1^st^-order BRNs and its central terminals forming the synapse with the 2^nd^-order barosensitive neurons in NTS. By using whole-cell patch techniques conjugated with intact nodose slice and horizontal brainstem slice, as well as the fluorescent dye labeling technique, the effect of ketamine on afferent conduction (CV) and excitatory post-synaptic currents (EPSCs) were evaluated. These data will provide a solid evidence to fully understand a likely cardiac dysfunction mediated by ketamine in its clinical application.

## RESULTS

### Time-dependent blocks of ketamine on afferent conduction of Ah-type BRNs in NG

**Figure 1:**
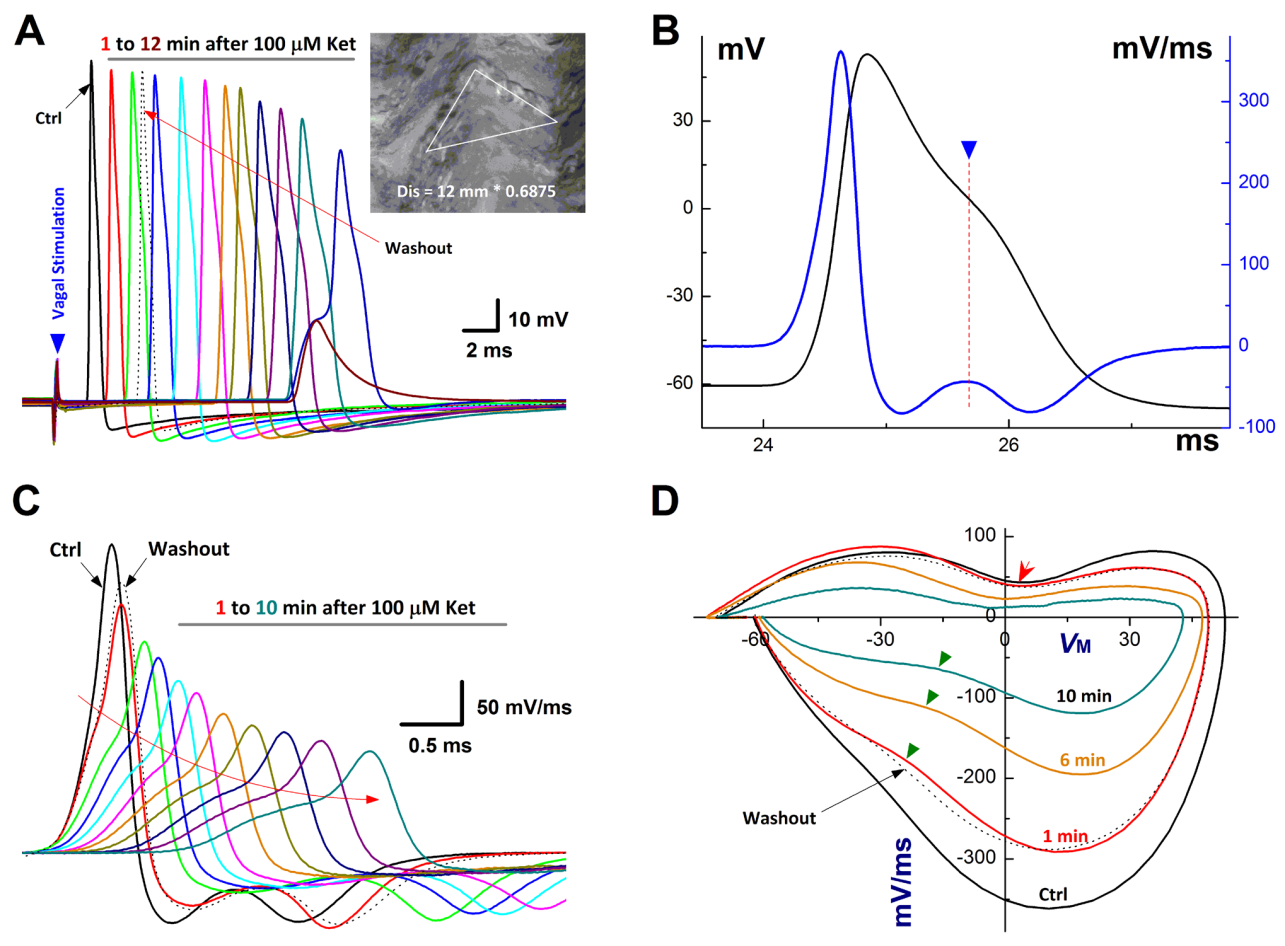
Time-dependently inhibition of Ketamine (Ket) on afferent conduction of Ah-type BRNs using intact nodose slice of female rats The AP was evoked by vagal stimulation (▼) and 100 μM Ket was applied by the bath perfusion. **A.** Vagal stimulation elicited AP before (Ctrl, black trace) and 12 min duration (colored traces) after 100 μM Ket and washout (black dotted trace) as well, *inset*: nerve fiber dissertations of Vagus after enzymatic treatment and the calibration to get the actual length for calculating the CV. The calibration factor for this nerve is 0.685 (shorter / longer) and the actual length for this nerve: 12 mm (distance between the electrodes) / 0.685 = 17.518 mm, the latency (the time from stimulus artifact to vagal evoked AP) is 1.67 sec, so the CV for this nerve is 17.518 mm / 1.67 = 10.48 M/s, suggesting that this is myelinated afferents; **B.** Ctrl AP evoked by vagal stimulation and the derivative calculated over the time course that plotted as the functional membrane voltage. By expended time scale, there is a clear repolarization hump over the time course of AP and the derivative (▼ and dot line), suggesting that this afferent neuron is the low-threshold and sex-specific subpopulation of Ah-type afferents; **C.** Vagal stimulation evoked AP before and 10 min after Ket; **D.** the displacement currents of phase plot: the derivatives were plotted as the functional membrane voltages.

In NG of adult male rats, both afferent conduction and discharge profiles of unmyelinated C-type afferents were significantly altered by ketamine but not myelinated A-types [4]. However, it remains unclear whether the neurons from age-matched female rats also respond the same way to ketamine, especially the sex-specific subpopulation of myelinated Ah-types. In this regard, the intact nodose slice [17, 21] was selected and vagal stimulation-evoked afferent conduction was tested before and after ketamine (Figure 1). Not surprisingly, in total 86 successful recordings from female nodose slices (*n* = 11 preparations), A- (*n* = 11, 12.79%) and C-type afferents (*n* = 63, 73.25%) responded the same way as the males to ketamine (data not shown). In stark contrast to A-types, even though Ah-types (*n* = 9, 10.46%) were myelinated afferents, the CV (11.78 ± 3.46 m/s) was dramatically and reversely inhibited in a time-dependent fashion by 100 M ketamine (Figure 1A). Averaged conduction blocks were observed at 10.08 ± 3.24 min after being exposed to ketamine.

In order to fully understand the detail changes in AP waveform before and after ketamine, the derivatives over the membrane potential and the displacement current phase plots [21, 29] were introduced, by which the insightful information regarding depolarizing (negative loop) and repolarizing (positive loop) phases of AP, as well as the relation of them could be viewed directly. Interestingly, during the time-dependent inhibition of ketamine, the UV_MAX_ and DV_MAX_ revealed by derivatives (Figure 1C) and the displacement currents phase plots (Figure 1D) were inhibited as the similar manner over the time course of AP. Whereas, the repolarization hump, the one of key feature to distinguish Ah- from A-types except for CV, remained (Figure 1D, indicated as red arrow). Clearly, the course of depolarization was smoothness before ketamine (Black trace), suggesting that the tetrodotoxin-resistive (TTX-R) Na^+^ currents kick in on time, right before the peak activation of tetrodotoxin-sensitive (TTX-S) Na^+^ channels. Whereas, in the presence of ketamine, the voltage-dependent activation for TTX-R was delayed, i.e., the TTX-R Na^+^ currents kick in after the maximal activation of TTX-S, which causes a separation of these components during the depolarization (Figure 1C, curved arrow; Figure 1D, indicated by ▼) into two functional portions. Averaged percentage reduction at each time points were no difference between these two portions (Supplemental Table 1), suggesting an equivalent inhibitory effect of ketamine on TTX-S and TTX-R components functionally expressed on myelinated Ah-type baroreceptor afferents and an equal contribution of these two components to AP depolarization and neuroexcitation. Obviously, the effects of ketamine on TTX-S and TTX-R Na^+^ currents may differ in Ah-types from either TTX-S in A-types or TTX-R in C-types in our previous investigation demonstrating that TTX-R is more susceptible to ketamine compared with TTX-S component in male rats [4].

**Figure 2:**
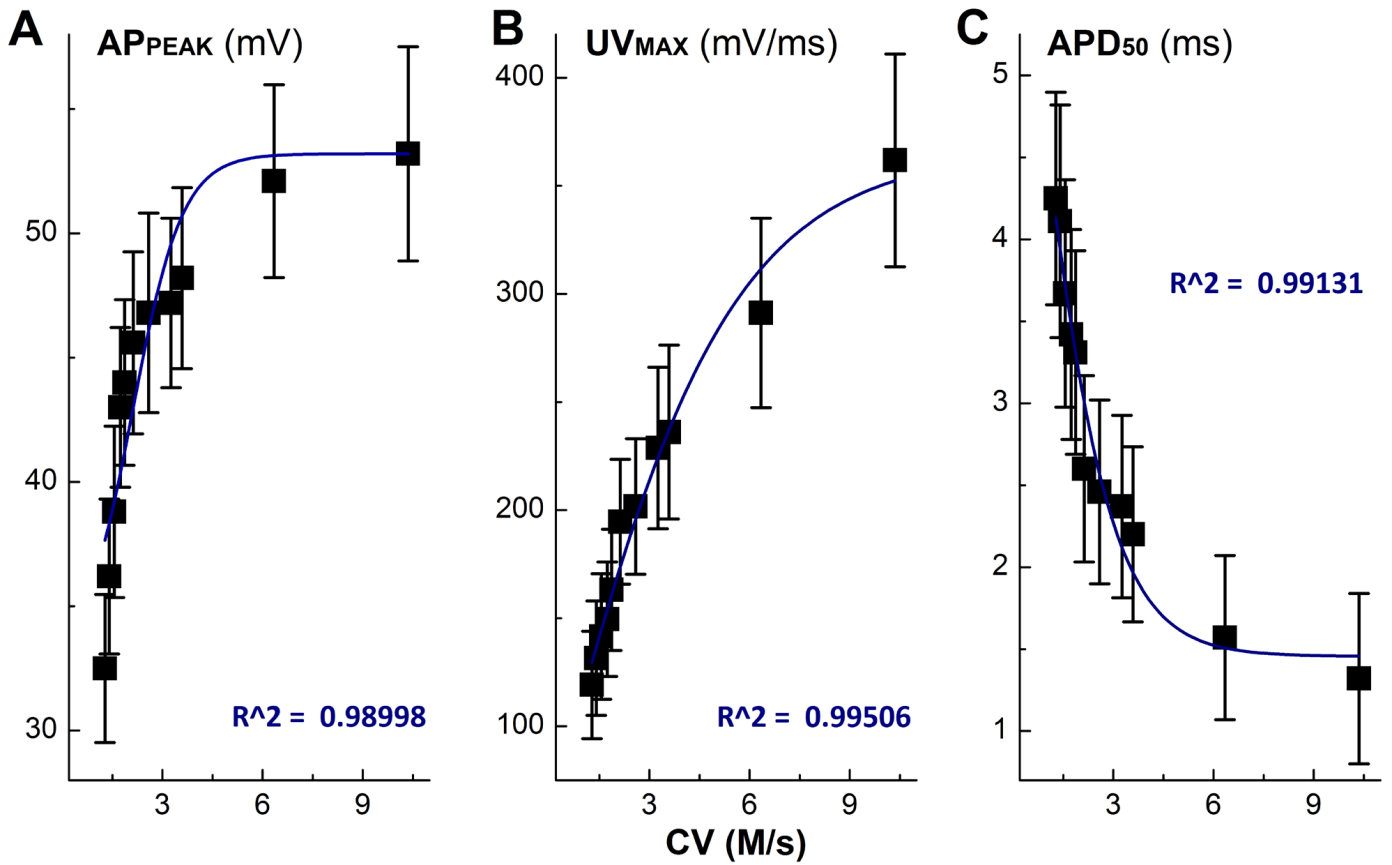
Non-liner relationships between the inhibitory effects of Ketamine (Ket) and AP discharge characters such as the peak of AP (AP_PEAK_), rate of depolarization (UV_MAX_), and AP duration (APD_50_)

Apart from the depolarization alternation along with the time-dependent CV blocks, the peak of AP (AP_PEAK_) was also reduced gradually with APD_50_. By plotting AP_PEAK_, APD_50_, and UV_MAX_ as the function of concentration of ketamine using a Boltzmann Equation, non-liner relationships were observed (Figure 2A-2C) between these functional parameters of AP depolarization and the concentration of ketamine, suggesting that at least two ion channel components are blocked by ketamine, which may closely be associated with AP depolarization. This observation was consistent well with the changes in derivative alternations over the membrane voltage showing the reduction in UV_MAX_ in the presence of ketamine (Figure 1C-1D).

### Concentration-dependent inhibition of ketamine on discharge profiles of Ah-type BRNs in NG

**Figure 3:**
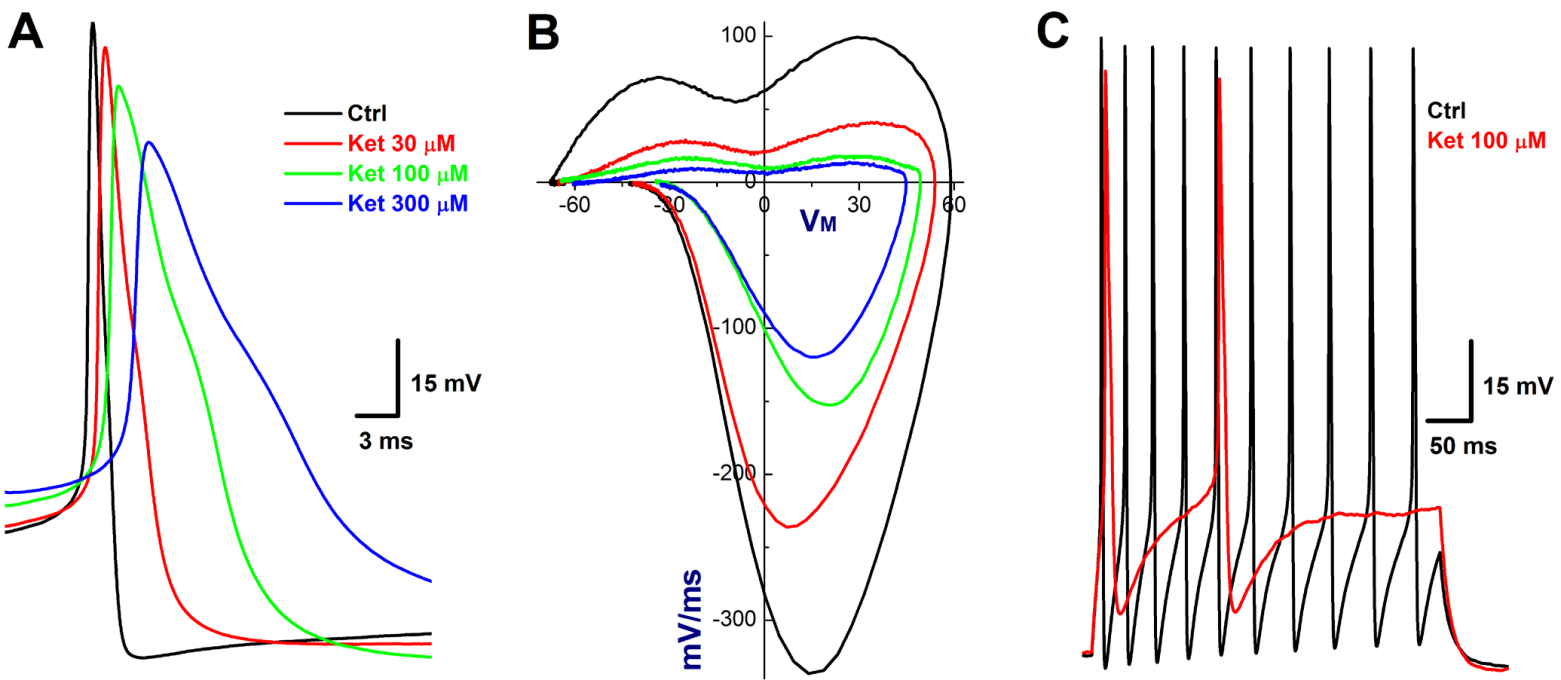
Concentration-dependent inhibition of Ketamine (Ket) on discharge profiles of Ah-type BRNs isolated from adult female rats Both single and repetitive discharges were elicited by brief pulse and depolarization steps. By measuring the AP threshold (APFT) and upstroke velocity conjugated with the repolarization hump, the afferent was identified as Ah-type BRNs. **A.** AP before and after 30 – 300 μM Ket; **B.** the derivatives before and after 30 – 300 μM Ket; **C.** the repetitive discharge before and after 100 μM.

In order to accurately evaluate the concentration-dependent profile of ketamine, the neurons were isolated from NG of adult female rats, which would be the best model for pharmacological investigation because the tested neurons could be completely and immediately exposed to the ketamine and washed out. However, due to the incapability to measure the CV, the afferent type of these isolated neurons need to be classified using electrophysiological validations [22] before ketamine. With this preparation, 11 neurons (11.34%) out of 97 successful recordings from 4 preparations matched the Ah-category and the percentage of distribution for Ah-type BRNs was similar to that being observed in the intact nodose slice and consistent well with our previous observation [17]. In the presence of ketamine, the maximal inhibitory effects were appeared at less than 2 min, which is sooner than those observed in intact nodose slice (∼10 min), and obviously, all discharge parameters (Supplemental Table 2) were significantly inhibited by ketamine in a concentration-dependent manner. By looking insight into the details of AP discharge characters, such as the reduction in the AP_PEAK_, significant depolarization in APFT (the take off point of membrane depolarization, Figure 3A), and dramatic decrease in UV_MAX_ (the negative peaks of displacement current phase plots, Figure 3B), ketamine-induced Na^+^ channel inactivation was highly suspected, meanwhile, the changes in APD_50_ and DV_MAX_ (the positive peaks of displacement current phase plots, Figure 3B) implied the inhibition of K^+^ channels (Figure 3A-3B), leading to the significant reduction in the frequency of repetitive firings (Figure 3C).

### Inhibitory effects of ketamine on EPSCs of Cap-insensitive Ah-type barosensitive neurons in NTS

**Figure 4:**
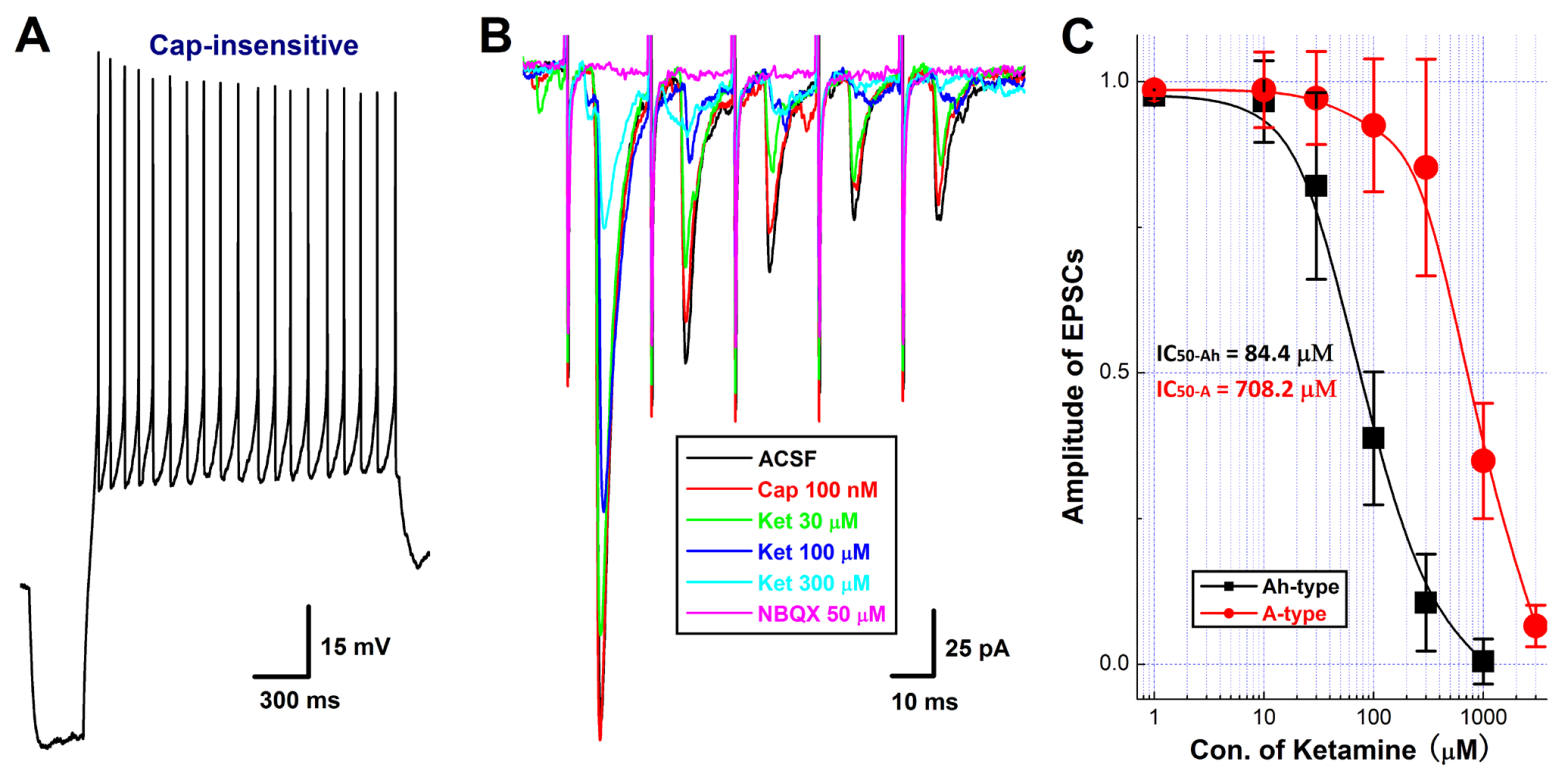
Concentration-dependent inhibition of Ketamine (Ket) on EPSCs of identified 2^nd^-order capsaicin (Cap)-insensitive and Ket-more sensitive Ah-type NTS barosensitive neurons in brainstem slice of adult female rats In this experiment, the AP was collected from fluorescent labeled, Cap-insensitive, but Ket-more sensitive 2^nd^-order NTS barosensitive neurons right before the EPSC was evoked by the solitary track stimulation using the protocols described in details in method section and supplemental materials. **A.** the AP elicited from Cap-insensitive Ah-type NTS neuron; **B.** the EPSCs in ACSF (black), 100 nM Cap (red), in the presence of 30 – 300 μM Ket (green, blue, light blue), and 50 nM NBQX (pink) collected from the same neuron shown in **A.**; **C.** the concentration-response relationship of Ket. The Cap-insensitive Ah-and Cap-insensitive A-type types (recordings shown in Figure. 6) were differentially blocked by Ket and the IC_50_ for Ah- and A-types were estimated at 84.4 μM and 708.2 μM, respectively.

**Figure 5:**
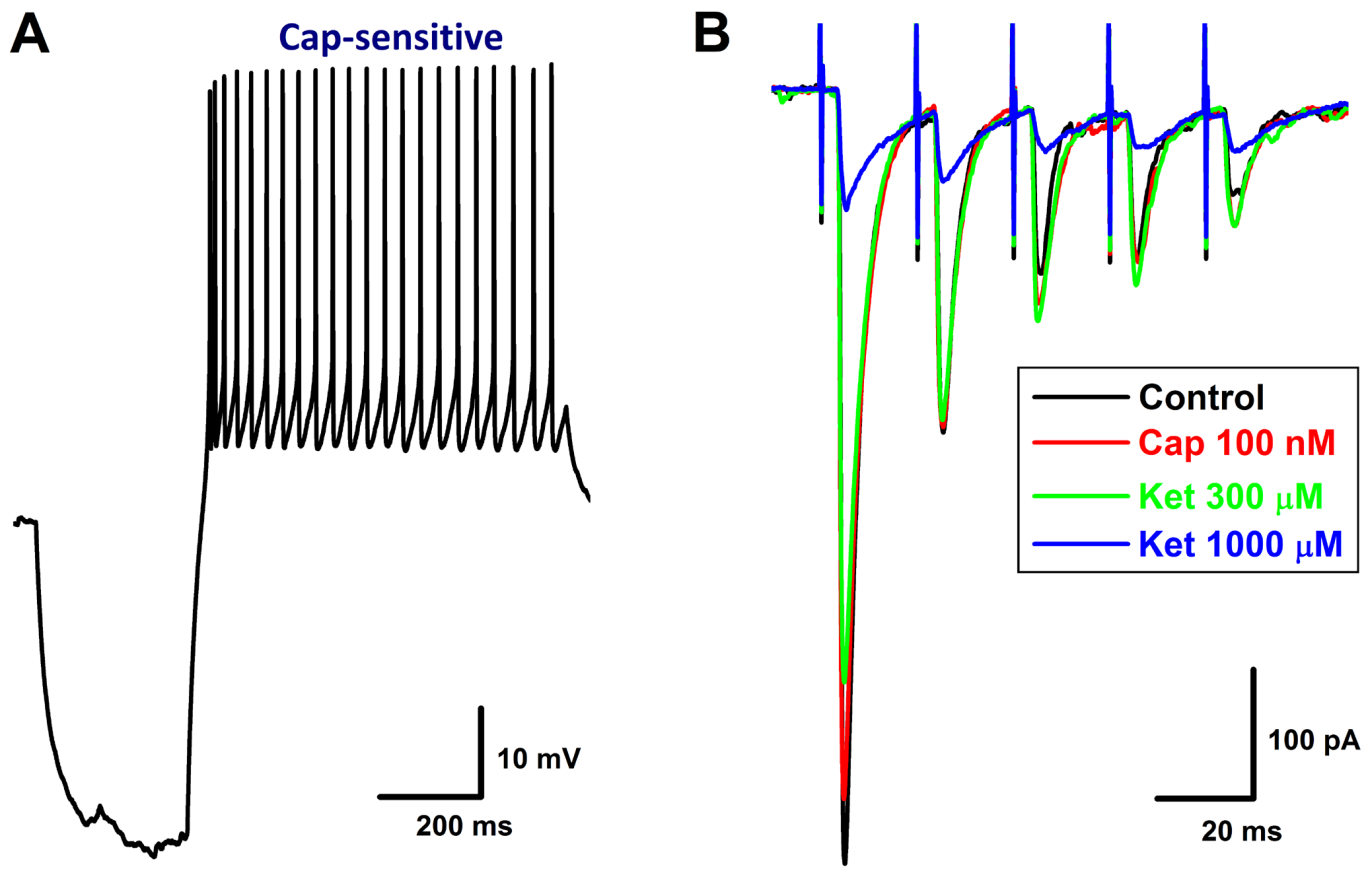
Concentration-dependent inhibition of Ketamine (Ket) on EPSCs of identified 2_nd_-order capsaicin (Cap)-insensitive A-type NTS barosensitive neurons in brainstem slice of adult female rats In this experiment, the AP was collected from fluorescent labeled, Cap-insensitive, but Ket-less sensitive 2^nd^-order NTS barosensitive neurons right before the EPSC was evoked by the solitary track stimulation using the protocols described in details in method section and supplemental materials. **A.** AP elicited from Cap-insensitive A-type NTS neuron; **B.** the EPSCs in ACSF (black), 100 nM Cap (red), and in the presence of 300 – 1000 μM Ket (green and blue) collected from the same neuron shown in **A.**

Even though the afferent conduction block was observed in the cell body of Ah-type 1^st^-order BRNs housed in the NG, the presynaptic transmission block may also be detected on their terminals synapsed with the 2^nd^-order barosensitive neurons located within the NTS based upon the never mixed principle of afferent transmission. To answer this particular question, the horizontal brainstem slice preparation was employed and the ST-stimulated EPSCs were recorded with or without ketamine. With this preparation, Cap-insensitive 2^nd^-order barosensitive neurons (*n* = 17) were identified from 9 NTS brainstem slices. Interestingly, total 8 (∼47%) of them (Figure 4A) were more sensitive to ketamine compared with the rest of observations (*n* = 9, Figure 5A-5B). The amplitude of EPSCs were reduced concentration-dependently by ketamine and the half maximal inhibitory concentrations (IC_50_) were estimated as 84.4 μM and 708.2 μM, respectively, for ketamine-more sensitive (presumable Ah-types) and ketamine-less sensitive (A-types) groups (Figure 4B-4C). However, EPSCs recorded from all Cap-insensitive barosensitive neurons in NTS were abolished by 50 μM NBQX (Figure 4B, pink trace) although they responded differently to ketamine.

**Figure 6:**
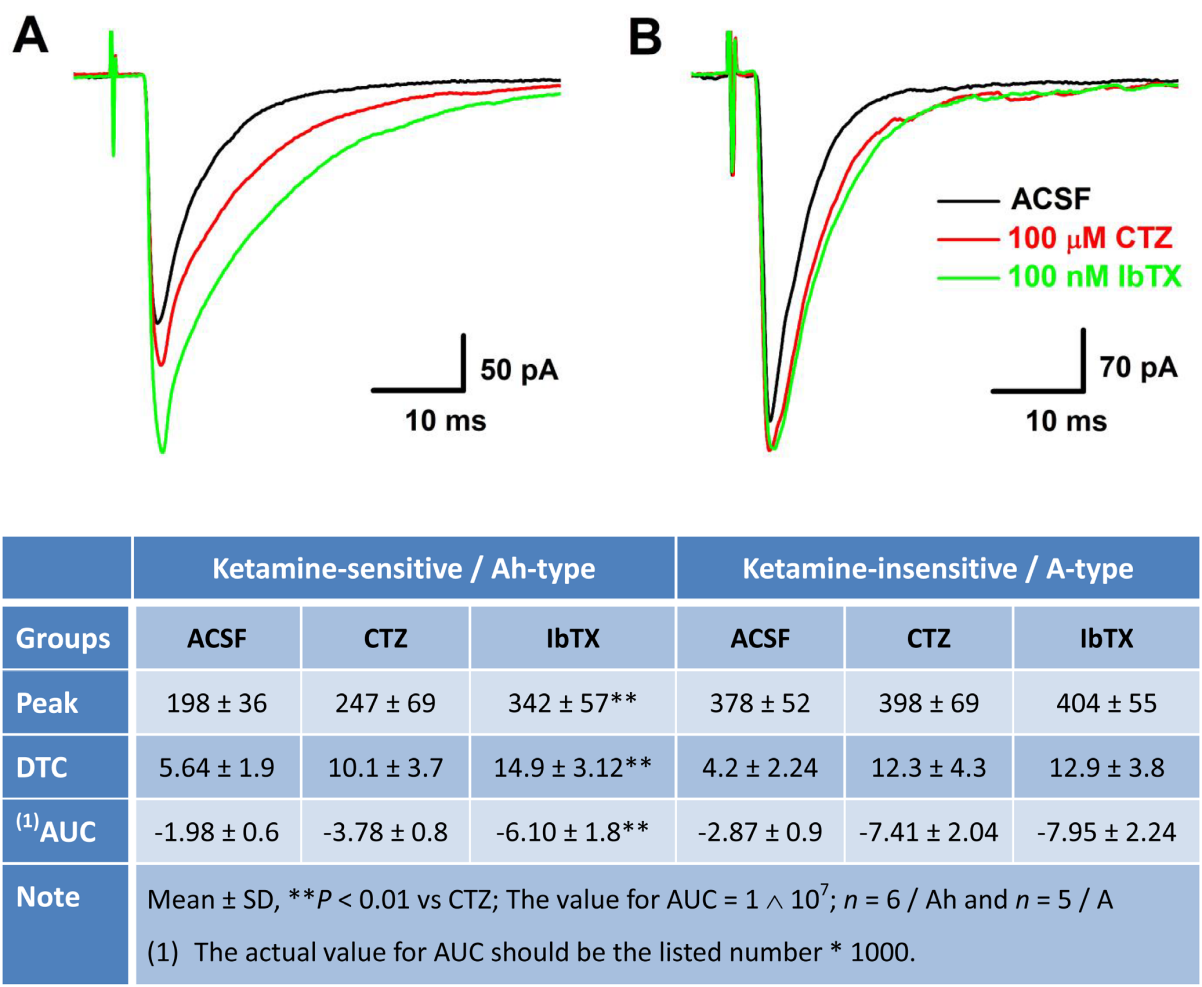
Differential effects of Iberiotoxin (IbTX) on the amplitude, decay time constant (DTC), and area under the curve (AUC) of EPSCs in ketamine (Ket)-more sensitive and Ket-less sensitive 2^nd^-order barosensitive neurons of NTS Even though these NTS neurons displayed a differential sensitivity to Ket, they were all capsaicin (Cap)-insensitive myelinated afferents, presumably mixed with myelinated A- and Ah-types, as those were observed in nodose ganglia. If this is the case, inactivation of KCa1.1 would change the dynamics of solitary track stimulation-evoked EPSCs of Ket-more sensitive rather that Ket-less sensitive group. **A.** and **B.** The changes in the amplitude, DTC, and AUC of EPSCs in ACSF, with 50 μM cyclothiazide (CTZ), and CTZ plus 100 nM IbTX, respectively, in Ket-more sensitive and Ket-less sensitive 2^nd^-order barosensitive neurons of NTS. The averaged dynamic parameters of EPSCs were summarized in the bottom table.

Recent observation [28] suggests that myelinated (Cap-insensitive) Ah-type barosensitive neurons from NTS respond to selective KCa1.1 blocker, iberiotoxin (IbTX), differently compared with myelinated A-types (Cap-insensitive), this may allow us identifying Ah-type afferents in the 2^nd^-order NTS neurons. After completely recording with ketamine, the most of tested Cap-insensitive neurons (*n* = 6 for ketamine-more sensitive, *n* = 5 for ketamine-less insensitive) were in a good condition and could be washed out at least partially with ACSF within 5 min. Even though these NTS neurons display a differential sensitivity to ketamine (Figures 4 & 5), they are all Cap-insensitive myelinated afferents, such as myelinated A- and Ah-types, as those are observed in NG. If this is the case, inactivation of KCa1.1 would alter the dynamics of EPSCs via upregulation of glutamate release from the presynaptic terminals in ketamine-more sensitive Ah-types rather than ketamine-less sensitive A-types [28]. As expected, after removing the fast desensitization at post-synaptic terminals with 50 μM CTZ, the averaged amplitude, decay time constant, and the area under the curve of EPSCs were all increased in the presence of 100 nM IbTX in ketamine-more sensitive (Figure 6A & bottom table), but not in ketamine-less sensitive group (Figure 6B & bottom table), implying that Cap-insensitive and ketamine-more sensitive neurons function as the 2^nd^-order myelinated Ah-type neurons in NTS and share exact same features as the 1^st^-order Ah-type in NG for the fast conduction [18] and functional expression of KCa1.1 [28], whereas, those neurons insensitive to Cap and less-sensitive to ketamine are traditionally classified as A-type of NTS [4].

### AMPA receptor expression in NG and NTS

**Figure 7:**
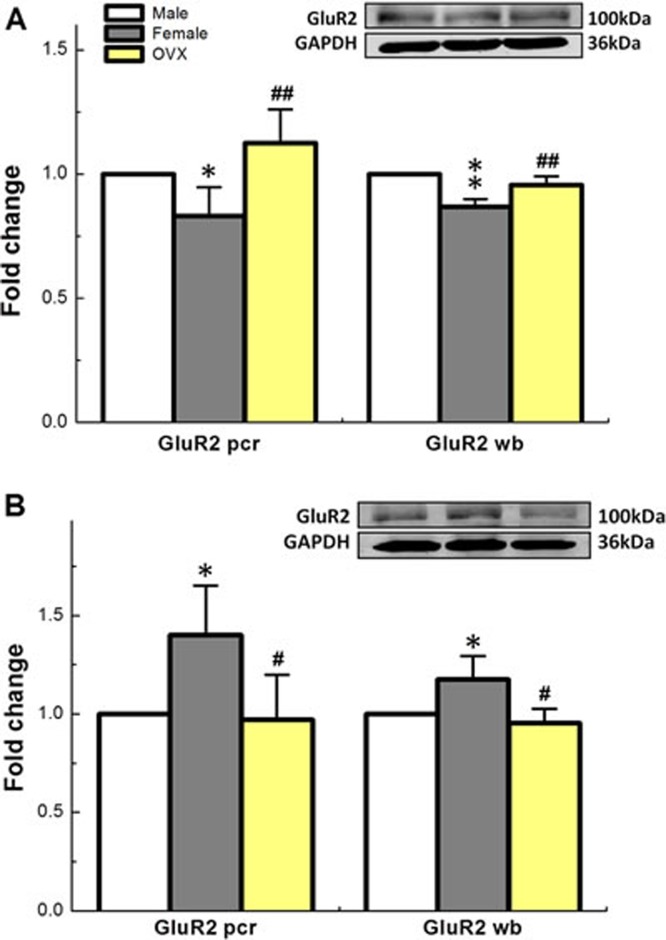
Sex-specific difference in expression profiles (fold change) of mRNA and protein for GluR2 receptor, one of subtypes of AMPA family, in NTS **A.** and NG **B.** of adult male, age-matched female, and OVX. Averaged data were expressed as mean ± SD. NTS: *n* = 4 rats (repeat for 3 times for both mRNA and western blot), and NG: *n* = 4 rats for mRNA and *n* = 9 rats for western blot (repeat for 4 times for both mRNA and western blot). *P < 0.05 and **P < 0.01 *vs* male, ^#^*P* < 0.05 and ^##^*P* < 0.01 *vs* female.

The α-amino-3-hydroxy-5-methyl-4-isoxazolepropionic acid receptor (AMPA) is a non-N-methyl-D-aspartate (non-NMDA) type ionotropic transmembrane receptor for glutamate that mediates fast synaptic transmission in the central nervous system and assembles from 4 genetically distinct subtypes including Glu1-4 [30]. NBQX has been reported to selectively block the excitatory actions of AMPA in rat [31]. Upon our electrophysiological and pharmacological tests with NBQX, the involvement of AMPA receptor in ketamine-mediated presynaptic transmission blocks of the 2^nd^-order Ah-type barosensitive neurons is highly expected. To test this hypothesis, the qRT-PCR and western blot were carried out in both NG and NTS. These data showed that GluR1 and GluR2 were detected in both NG and NTS, however, females expressed significant less mRNA and protein for GluR2 in NTS compared with age-matched males and the expression profile was upregulated in ovariectomized (OVX) females (Figure 7A). Intriguingly, the expression patterns for both mRNA and protein of GluR2 in NG were in stark contrast to those in NTS (Figure 7B). Even though the difference of NG and NTS expressed GluR1 did not established between sexes (data not shown). These molecular data suggest that the female hormone-dependent expression of GluR2 receptor in NG and NTS plays a critical role in sexual-dimorphisms, at least partially, in sex- and afferent-specific presynaptic neurotransmission of baroreflex afferent function and ketamine-mediated cardiovascular dysfunction.

## DISCUSSION

### Myelinated Ah-type barosensitive neurons in NTS

The major finding of this study is the ketamine-mediated afferent-specific conduction blocks in low-threshold and sex-specific subpopulation of myelinated 1^st^-order Ah-type BRNs identified by conduction velocity [21, 22] in NG and presynaptic neurotransmission blocks in ketamine-more sensitive myelinated 2^nd^-order Ah-type barosensitive neurons identified by Cap- [4] and IbTX- sensitivity [28] in NTS.

### Potential role of Ah-type baroreceptor neurons of NG and barosensitive neurons of NTS in physiological and disease condition

This is the first direct evidence to demonstrate that Ah-type neurons in NG or Ah-type barosensitive neurons in NTS are much more sensitive to ketamine compared with A-types, even though they are all Cap-insensitive and myelinated afferents. Simply because of the female-specific distribution of the Ah-type neurons [17], the current observation highly suggests that ketamine-mediated presynaptic transmission blocks may not only contribute to the cardiac pathophysiological processes, including autonomic nerve and baroreflex function [4-7, 32], pro-arrhythmic effect and problematic rise in blood pressure [2, 8-10, 32], ventricular remodeling [13], cardiac arrest [14], but also be responsible for the potential gender difference in cardiac function [3] in clinical management with ketamine. Therefore, more attention needs to be emphasized on all aspects in clinical practice with ketamine in those patients with ischemic heart diseases [33]. Importantly, more investigation regarding ketamine needs to be conducted using female species at the bench and special awareness has to be focused on the female patients at the bedside during ketamine application to enhance our understanding in knowledgebase of ketamine. Moreover, except for the sex-specific distribution characteristics, Ah-type BRNs in NG or Ah-type barosensitive neurons in NTS share both features of myelinated afferents, like fast conduction [18, 22] and Cap-insensitivity [23], and unmyelinated afferents, such as ion channel expression [18, 28] and chemosensitivity to histamine [24], so that it may play an important role not only in regulating the mean blood pressure [34, 35] but also in buffering the dynamic changes in blood pressure [36-38], which are the major functions for C- and A-type baroreceptor afferents, respectively.

### Collaborative contribution of TTX-S and TTX-R Na^+^ channels in Ah-type baroreceptor afferents

The afferent conduction block of ketamine observed in the cell body of the 1^st^-order neurons is a time- and concentration-dependent, which is attributed at least to the inhibition of voltage-gated Na^+^ channels [39, 40], whereas, the presynaptic transmission block-mediated by AMPA and NMDA receptors is a central mechanism of ketamine at afferent terminals of the 1^st^-order neurons [4, 41, 42]. Voltage-gated Na^+^ channels (Nav1.7 and Nav1.8) are critical for afferent conduction, and the current observation showed that both Nav1.7 (TTX-sensitive, TTX-S) and Nav1.8 (TTX-resistant, TTX-R) are both involved in conduction in Ah-type afferents, which could be blocked by ketamine leading to the conduction failure of vagal and solitary track neurotransmission revealed by the present (Figure 1A and Figure 4B) and previous observation [4]. However, several questions remain to be answered. Firstly, whether TTX-S and TTX-R Na^+^ channels play an equal role in afferent conduction; secondly, it is not clear what the relationship is to mediate afferent conduction between TTX-S and TTX-R Na^+^ channels; additionally, if TTX-S or TTX-R Na^+^ channel alone evokes the conduction. Interestingly, the intact nodose slice preparation [39] would be a proper way to answer the above questions.

### The equal contribution and cooperation of TTX-S and TTX-R Na^+^ channels in AP formation and afferent conduction of Ah-type neurons in baroreflex afferent pathway

**Figure 8:**
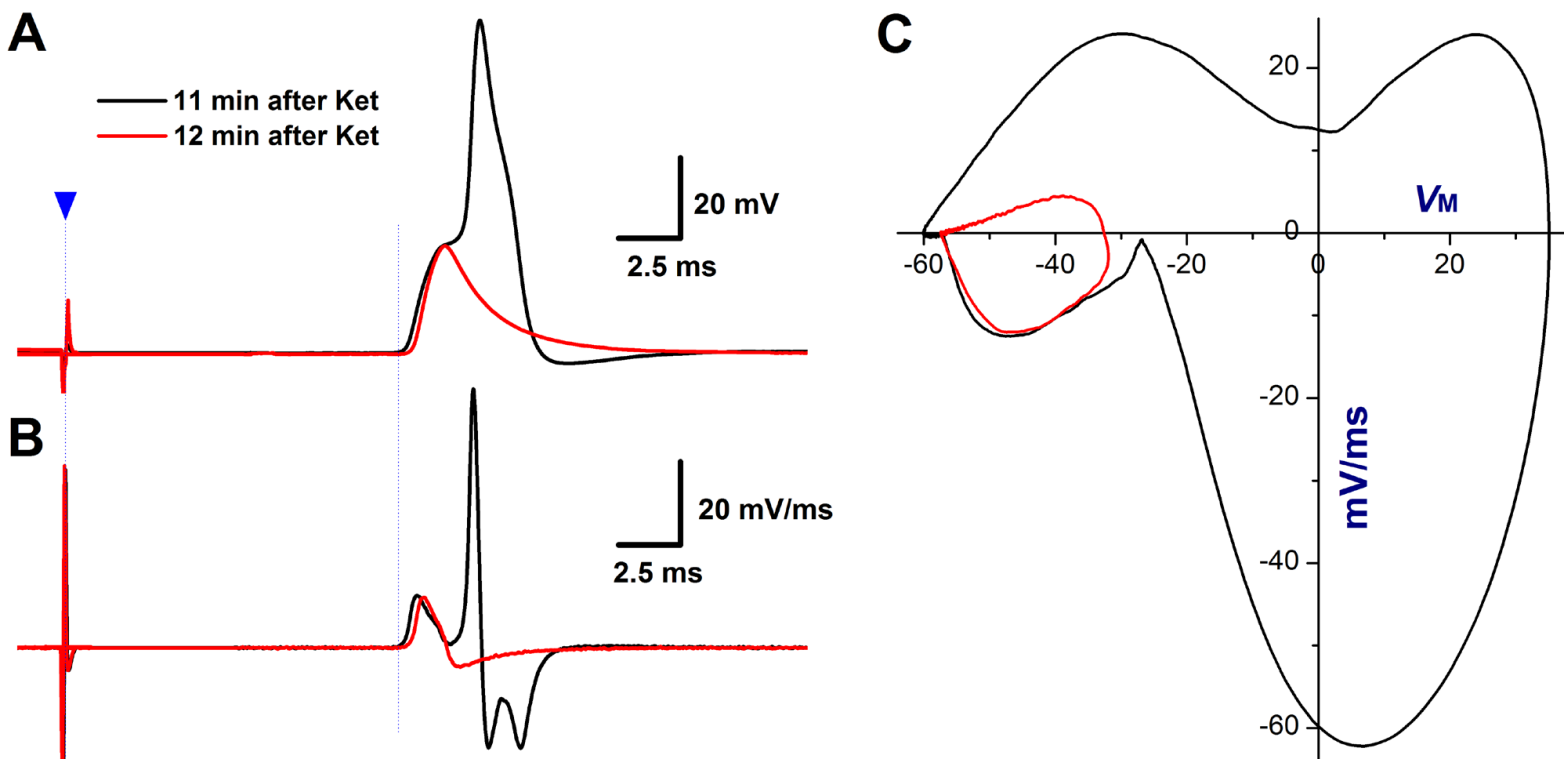
TTX-S and TTX-R components of Na^**+**^ currents are both involved in afferent conduction and action potential (AP) formation of Ah-type BRNs in intact nodose slice of adult female rats As shown in the figure 1, vagal stimulation-evoked APs were collected at 11-12 min after 100 μM Ketamine (Ket). **A.** AP dissertation was observed at 11 min after Ket due to the delay activation of TTX-R component, which was completely blocked by Ket at 12 min after application with remaining TTX-S component; **B.** the derivatives over the membrane potential plotted as the function of membrane voltage from traces shown in **A.**, and clear separation of TTX-S and TTX-R components of Na^+^ currents was observed at 11 min after Ket, and remaining TTX-S component alone at 12 min after Ket; **C.** the separation of TTX-S and TTX-R components was also confirmed by the displacement current phase plots.

The derivatives over the membrane potential and displacement current phase plots are two useful methods for quantification of TTX-S and TTX-R Na^+^ channel contribution and cooperation in AP formation and afferent conduction [21, 29]. Clearly, in the control condition, the smooth derivatives of depolarization in vagal stimulation-evoked AP (Figure 1B) imply that the TTX-R component recruits before the TTX-S component reaches the maximal. In contrast, in the presence of ketamine, the amplitude and slope in rising phase of derivatives reduces markedly with significantly lesser smoothness (Figure 1C, as indicated by curved arrow), suggesting that both TTX-S and TTX-R components are partially inhibited with equal potency (Supplemental Table 1) and TTX-R Na^+^ currents kick in after TTX-S component reaches the maximal before conduction failure, this phenomenon is also confirmed by the displacement current phase plots (Figure 1D, indicated by green arrow heads). Interestingly, the significant plateau (Figure 8A, black) in AP depolarization is observed right before conduction failure with corresponding separation of TTX-S and TTX-R components revealed in derivatives (Figure 8B) and the displacement current phase plots as well (Figure 8C). In this circumstance, reduced TTX-S still induces membrane depolarization that barely reaches the threshold for TTX-R component and leads to the distorted AP, suggesting a key role of TTX-S in initiated AP formation and propagation.

Further incubation with ketamine, the TTX-S remains but TTX-R component disappears (Figure 8A-8C, red). These data highly suggest that TTX-S coordinates closely with TTX-R during AP depolarization, and there is clear difference in activation threshold for both TTX-S and TTX-R component and TTX-S is an essential for TTX-R activation simply because if TTX-S activation could not make the membrane depolarized to the threshold for TTX-R in the presence of ketamine, then the conduction failure would absolutely occurred even though TTX-R component may still be available for activation. In another word, the TTX-S component in identified low-threshold and sex-specific myelinated Ah-type BRNs housed in NG is critical for setting the firing threshold, while, TTX-R component is a key player for further depolarized membrane to form a propagated AP.

From this observation, the conclusion could not be drawn if TTX-S is less sensitive to ketamine compared with TTX-R component even though it disappears later than TTX-R, which may be masked by significant reduction in TTX-S component by ketamine leading to less membrane depolarization up to the threshold for TTX-R, in this case, even if there is enough TTX-R available it would remain not to be activated, so the explanation from this observation will not be contradicted with literature [39]. From the cellular point of view, Ah-type afferents not only express similar ion channels compared with the C-type afferents, including TTX-S/TTX-R [18, 27], KCa1.1 [28, 43], and HCN1 [19, 20, 44], but also show identical chemosensitivity to histamine [24, 25], so, it is not surprised that Ah-type afferents display a similar reaction to ketamine within presynaptic neurotransmission on the cell body and synaptic terminal of 1^st^-order neurons as indicated in C-type afferents [4]. Although the effect of ketamine was not evaluated in the observation, the significant slow repolarization and broader AP duration suggested that certain K^+^ channels must be blocked by ketamine, which is consistent with the previous documentation [40, 45]. Moreover, conjugated with our intact nodose slice data and the data collected from isolated neuron preparation, we also conclude that afferent conduction definitely failed before somatic AP generation because AP can still be elicited in isolated Ah-type BRNs with higher concentration of ketamine causing the presynaptic neurotransmission failure.

Upon the establishment of intact nodose slice preparation [17, 21, 22], this low-threshold and sex-specific subpopulation of myelinated Ah-type baroreceptor afferents have extensively been investigated for more than a decade. Due to their female-dependent distribution, relatively high neuroexcitability [17, 19, 28, 44, 46] with female hormone-dependent, the A-type-like fast afferent conduction, and C-type-like electrophysiological and chemosensitive properties to histamine and IbTX, so, Ah-type afferents may play a great contribution in baroreflex afferent function to elicit larger blood pressure and heart rate change like A-types to buffering dynamic changes in pressure [34, 35] and provide powerful antihypertensive baroreflex responses like C-types to regulate mean blood pressure [36-38], which would retain a relative a low blood pressure in female rats compared with age-matched males [26]. Together with the data collected from the present experiments, we have a strong reason to believe that ketamine-mediated presynaptic neurotransmission blocks would cause dramatic cardiovascular dysfunction via baroreflex afferent pathway [32], especially in those female patients with or without cardiovascular diseases.

## MATERIALS AND METHODS

### An expanded methods section is available in the online-only data supplement

All animal procedures and protocols were performed with the pre-approval of the Institutional Animal Care and Use Committee at Harbin Medical University, which are in accordance with the recommendations of the Panel on Euthanasia of the American Veterinary Medical Association and the National Institutes of Health publication “Guide for the Care and Use of Laboratory Animals (http://www.nap.edu/readingroom/books/labrats/).”

### Experimental animals

Adult female Sprague Dawley (SD) and aged-match male rats (240-280 g) were selected for the molecular experiments, the preparations of intact nodose slice, isolated neurons, and horizontal brainstem slice. Rats were directly purchased from Wei-Tong-Li-Hua Experimental Animal Technology Co, Ltd, Beijing, China, with SPF grade and licensed under SCXK (Beijing) 2012–0001. All rats were maintained at the animal facility of the Second Affiliated Hospital of Harbin Medical University with a 12/12 hour light cycle for 3 days before they were used for experiments. Ovariectomized (OVX) female rats were also used to evaluate the hormone-dependency of expression profiles of NMDA type-I receptor in NG and NTS. The protocol for surgery procedures of OVX was described in detail elsewhere [44].

### Chemicals

Capsaicin (Cap, a TRPV1 agonist), cyclothiazide (CTZ, a modulator of AMPA receptor for removing rapid desensitization at postsynaptic terminals), 2,3-Dihydroxy-6-nitro-7-sulfamoyl-benzo(F) quinoxaline (NBQX, antagonist for non-NMDA), and Iberiotoxin (IbTX, a selective blocker for a large conductance of Ca^2+^-activated-K^+^ channel, KCa1.1) were purchased from Sigma (St Louis, MO, USA); Stock solutions were stored at −20°C and diluted using the bath solution right before experiments. During the experiment, drugs or toxins were applied through bath perfusion or micro-perfusion right on the patched neuron at flow rate no more than 1.0 ml/min.

### Intact nodose slice

The NG contains the cell body of afferent neurons (the 1^st^-order) that send afferent signals via the solitary track (ST) to form the synapses with the 2^nd^ order neurons in NTS. Using this nodose slice preparation, the action potential (AP) was evoked by vagal stimulation and afferent conduction velocity (CV) was also calculated to classify the afferent fiber type. The methodology of surgical procedures and tissue preparations for nodose slice are described in details in expended methods section (see online Supplemental Materials) or elsewhere [21, 22].

### Dil labeling

The lipophilic fluorescent dye (Dil, Molecular Probe) was used to label the aortic depressive nerve (ADN) in younger SD rats to confirm the 1^st^-order BRNs in NG and 2^nd^-order barosensitive neurons in NTS. The procedures of Dil labeling were described in details previously [47]. Briefly, the rats at 4 weeks of age (75∼100 g) were anaesthetized, the leftside ADN was dissected carefully near aortic arch, and then Dil crystals were placed on top of the ADN and sealed completetly to prevent Dil from contaminating the Vagus. At least one week after the survival surgery is necessary to allow the Dil to retrograded transport to NG and NTS. In this experiment, the adult SD rats were used to ensure the completely myelination, in this regard, at least 8∼10 weeks after labeling, the nodose slice or horizontal brainstem slices could be prepared for electrophysiological data collection

### Horizontal brainstem slice

The NTS contains the cell body of the 2^nd^-order neurons within visceral afferent pathway to integrate and relay the processes to higher regions of the brain. Horizontal brainstem slices of the NTS that included a sufficient length of the solitary tract to electrically evoke monosynaptic AMPA receptor-mediated currents in NTS neurons were prepared [4]. Initial synaptic characterization of NTS neurons was carried out using a burst of five 200 μs current pulses delivered at 50 Hz, with a three second interval between each trail. Stimulus intensity was gradually increased until a corresponding train of excitatory postsynaptic currents (EPSC) was recorded in the patched NTS neuron. Tract stimulus intensity was then increased beyond this threshold up to 5 times greater. Only those EPSCs exhibiting a jitter (standard deviation of latency) ≤ 200 μs and amplitude saturation in response to increases in stimulus intensity were considered to arise from monosynaptic, the 2^nd^-order NTS neurons and thus suitable for further study. The ST-evoked EPSCs were recorded and their dynamic changes, such as the amplitude, the delay time constant, and the area under curve of EPSCs were evaluated before and after ketamine in this brainstem slice preparation [4, 28].

### Isolated nodose neurons

In order to accurately evaluate the concentration-dependent property of ketamine, isolated nodose neurons were isolated enzymatically using adult rats according to the protocols described in detail previously [21, 29]. A single AP and repetitive discharge were elicited by a brief pulse and step depolarization current injection before and after ketamine.

### Fiber type identification for nodose neurons

In intact slice and at room temperature (22∼23°C), the afferent fiber types of nodose neurons were classified by CV, a fast conducted (CV > 4 m/s) without or with hump feature during the repolarization was identified as myelinated A- and Ah-types, whereas, a slow conducted (CV < 1.0 m/s) was identified as unmyelinated C-types [21].

For isolated neurons, a set of AP discharge characters, such as AP firing threshold, upstroke velocity, AP duration and repolarization hump, were used in combination for isolated neurons identification [22]. Briefly, A-type was characterized as low-threshold (APFT: > −40 mV) with the maximal up- (UV_MAX_: > 250 mV/ms) and down-stroke velocity (DV_MAX_: > −100 mV/ms), narrow AP duration (APD_50_: < 1.0 ms), a negative presentation of repolarization hump; Ah-type showed a similar APFT and UV_MAX_, and slower DV_MAX_ (−75∼100 mV/ms), wider APD_50_ (< 2.0 ms) with hump feature compared with A-type; while, for C-type, the APFT was more depolarized (−30 mV), both UV_MAX_ (< 200 mV/ms) and DV_MAX_ (< −50 mV/ms) were significant slower with much wider APD_50_ (> 2.0 ms) and dramatic repolarization hump. In addition, the chemosensitivity to capsaicin (Cap) was also useful in identification of the afferent type. Both A- and Ah-types were insensitive to 100 nM Cap, while the C-types showed a positive respond to Cap. Due to the difficulty to washout, Cap must be applied at the end of the recordings.

### Fiber type identification for NTS neurons

The afferent fiber types of the 2^nd^-order neurons of NTS were confirmed by sensitivity to Cap applied at the end of recordings and the feature of delay excitation [23, 48]. Both A- and Ah-type NTS 2^nd^-order neurons are Cap-insensitive without detectable delay excitation, but the property of EPSCs of Ah-types is altered by Iberiotoxin (IbTX), whereas, A-types are not [28]. In contrast, C-types are Cap-sensitive with clear delay excitation and IbTX-mediated a significant changes if the dynamics in the property of EPSCs.

### Western blot analysis for GluR2 protein

The animals were euthanized with an excessive dose of pentobarbital sodium. Both sides of the nodose ganglia and NTS were removed immediately and frozen on liquid nitrogen. Nine pairs of tissues from NG and 4 tissue samples from NTS were collected for Western blots. The tissues were lysed by using lysis buffer (Beyotime Biotech, China) containing 1% protease inhibitor solution (Beyotime). The lysate was centrifuged for 15 min to collect supernatant. The protein concentration was determined using BCA Protein Assay Kit (Beyotime). The samples were boiled for 7 min and then loaded on 10% SDS-PAGE gel (100 mg of protein, 15 ml per well) for electrophoresis with 110 V for 90 min. The protein on the gel was transferred onto NC membranes at 300 mA for 75 min, which was blocked by 5% nonfat dry milk diluted by PBS at room temperature for 3 hours. The membrane was probed with primary antibody (GluR2 receptor rabbit polyclonal antibody, Alomone Labs, 1:200) at 4°C overnight and then the secondary antibody (goat anti-rabbit, Licor, 1:8000) for 60 min at room temperature. GAPDH was used as the internal control. Bound bands were visualized and analyzed using Odyssey Infrared Imaging System (LI-COR Biosciences) and Odyssey v1.2 software.

### Real-time quantitative PCR for test GluR2 mRNA

For SYBR Green RT-PCR, 4 SD rats were used to harvest total mRNAs from NG and NTS in each group. Total RNAs were extracted using the TRIzol^®^ Reagent (Invitrogen) according to the manufacturer's instructions. The cDNAs were synthesized using the Reverse Transcription Kit (Applied Biosystems). Quantitative PCR reactions were run on an ABI 7500 fast Real-Rime PCR System (Applied Biosystems). The primers (Invitrogen) were used as follows: 5′-ATTGTAGACTACGATGATTC-3′ (forward) and 5′-AATAGTCAGCTTGTACTTGA-3′ (reverse) for GluR2. GAPDH was used as an internal control. The primer sequences of GAPDH were: 5′-AAGAAGGTGGTGAAGCAGGC −3′ (forward) and 5′-TCCACCACCCAGTTGCTGTA-3′ (reverse). The 2^−ΔΔCt^ method was applied for the data analysis and the data were normalized and converted into relative mRNA expression.

### Data analysis

The recordings were acquired by Clampfit and the data were analyzed by using Excel, Origin and SPSS software. The figures were prepared with Origin. The paired *t*-test was selected for statistical analysis for the changes in afferent conduction and discharge profiles before and after ketamine. The EPSC data were averaged from 10 traces from one recording and compared before and after ketamine using *t*-test and ANOVA where appreciated. The mRNA and protein expression for NMDA receptor were expressed as the fold changes and *t*-test was used for the comparison between groups. An averaged data were expressed as mean ± SD. The *P* value less than 0.05 were considered significantly difference.

## SUPPLEMENTARY MATERIAL TABLES


