# Supplementary material for: Ketamine-mediated afferent-specific presynaptic transmission blocks in low-threshold and sex-specific subpopulation of myelinated Ah-type baroreceptor neurons of rats
Source: Oncotarget. 2015 Dec 12;6(42):44108–22. doi: 10.18632/oncotarget.6586 (PMC4792545; doi:10.18632/oncotarget.6586)
Supplement: Supplementary file 1 [file oncotarget-06-44108-s001.pdf]

Ketamine-mediated afferent-specific presynaptic transmission blocks in low-threshold and sex-specific subpopulation of myelinated Ah-type baroreceptor neurons of rats

Supplementary Material

Supplemental Digital Content - Expanded Methods

Surgical ovariectomy

The surgery was performed following protocols described in details previously.[1] Briefly, anesthetized animals (combination of xylazine 10 mg/kg and ketamine 75 mg/kg) were placed in a lateral position, and both flanks were shaved and cleaned using chlorhexidine scrub and disinfected with 70% ethanol and povidone-iodine (7.5%). A 2.0-cm incision was made on the left lateral side along a line spanning from the 2nd to the 5th lumbar vertebra, using a scalpel blade. The left ovary and associated fat were located and externalized by gentle retraction. After removal of the ovary, the peritoneal cavity, muscle layers, and skin were closed successively with 4-0 absorbable sutures and then penicillin (80000 Units) was given via intramuscular injection. The same procedure was repeated for removal of the right ovary. After recovering from anesthesia, the animals were monitored for at least 30 min to ensure that there was no bleeding from the surgery, and then were returned to the animal facility. Four weeks after ovariectomy, the animals were sacrificed for experimental use.

Aortic depressor nerve (ADN) labeling

The fluorescent labeling of ADN with dye DiI was performed and the procedures were described as previously reported.[2-4] Briefly, after the anesthesia with the cocktail mentioned above and proper treatments of the skin on the surgical area, a ~2 cm incision was made under aseptic conditions along the left ventral side of the neck. A blunt dissection of the underlying musculature exposed the left carotid artery and surrounding nerve fibers. Under higher magnification, the left ADN, which exclusively contains baroreceptor fibers arising from

the aortic arch, was identified. The ADN was separated from the Vagus and sympathetic nerves and placed in a 5 mm long sterile silicon trough. A few crystals of the lipophilic fluorescent dye DiI was placed on the ADN. The nerve, dye crystals and trough were coated with ~0.3 ml of a peripheral nerve encapsulant (Kwik-Sil, WPI). The area was rinsed with sterile saline and the skin was closed using vicryl suture. The animal fully recovered before returning to the animal facility. At least 4 weeks passed before the animal was considered fully healed and available for experimentation.

Isolated neurons for electrophysiology

Isolation procedure was described in details previously.[5-7] Briefly, after both sides of nodose ganglia were surgically removed they were cut into six pieces of each ganglion and then placed in cold nodose complete medium (NCM) composed of Mito™ + serum extender (v/v, 1:20, Collaborative Biomedical Products, Bedford, USA), Dulbecco's modified Eagle's medium F-12 (Gibco, Grand Island, USA) supplemented with 5% fetal bovine serum (HyClone, Logan, USA) and 1% penicillin-streptomycin-neomycin antibiotic mixture (PSN; Gibco, Grand Island, USA). The ganglia were transferred to an the 1st enzyme solution containing 20 units/ml of Papain (Worthington) in Earle's balance salt solution (Sigma, San Louis, USA) at 37°C for 20-22 min. The enzyme solution was replaced with the 2nd enzyme solution containing 2 mg/ml of Dispase II (Roche) and 1 mg/ml of Collagenase type-II (Worthington) in Earle's balance salt solution (Sigma) at 37 °C for additional 30-35 min depending upon the animal age. The enzyme solution was replaced with NCM containing 1.5 mg/ml albumin (Bovine, Sigma) and the ganglia were dissociated by trituration with fire-polished pipettes. Cell suspension was placed on poly-D-lysine (Sigma, San Louis, USA)-coated glass coverslips and cultured at 37°C moisture environment for at least 4 h before recording.

Whole-cell current- and voltage-clamp patch recordings[6, 7] were conducted using an Amplifier 200B or 700B (Axon Instruments, Union City, USA). Borosilicate glass pipettes (BF150-86-10; Sutter, Novato, USA) were

pulled (P-97, Sutter, USA) and polished (F-83, Narishige, Japan) down to a resistance of 1.2-1.8 MΩ. Following correction for all offsets, a giga-ohm seal was formed and the pipette capacitance was compensated. Total cell capacitance (30-50 pF) and electrode access resistance (3-5 MΩ) were also compensated (60-80%). Recordings were low pass filtered to 10 KHz and digitized at 50 KHz. Experimental protocols, data collection, and preliminary analysis were performed using pCLAMP 10.3 and Digidata 1440A (Axon Instruments, Union City, USA). Corrections for liquid junction potentials were taken into consideration before final data analysis. The single and repetitive AP firings elicited by brief pulse and step depolarization current injects were collected, respectively, from each tested neurons for neuronal identification and discharge profile analysis before and after applications.[7]

Vagus-nodose slice for electrophysiology

Slices of nodose ganglia with intact vagal axons were prepared in a manner previously described.[6, 7] Briefly, adult SD rats (250–300 g) of either gender were used for the slice preparation. The unrestrained rats were placed in an airtight induction chamber for inhalation of the anesthetic Metofane (Methoxyflurane, Schering-Plough Animal Health Corp, Kenilworth, NJ, USA). Upon lack of reflex response to tail pinch the animals were immediately sectioned at the mid-auxiliary region, preserving at least 2 cm of the Vagus nerve. The nodose ganglia with attached Vagus were excised under stereomicroscopy (×40). The tissue was immediately placed in chilled (4°C) recording solution. Slicing exposed the interior of the ganglion capsule and the tissue was placed in a solution of Earle's balance salt solution (Sigma) containing type II Collagenase (1.0 mg/ml) at 37°C for 40 – 45 min followed by the solution containing Trypsin-3X (5 mg/ml) for another 20 – 22 min. The tissue was moved to the bath perfusion chamber and allowed to recover for 1 h prior to recording. The identical whole-cell patch techniques were used as described above. After tightened seal, electric evoked currents by directly stimulation of Vagus[8] was collected under cell-attach configuration in AP bath solution with peptide solution

for Ca^{2+} current recording, and neuron type could be identified upon the afferent fiber CV. And then whole-cell Ca^{2+} currents could be collected by completely bath perfusion with Ca^{2+} current recording solution. Cell was held at −100 mV and voltage was stepped from−80 mV to +30 mV for 400 ms with 10 mV increments and 1 s interval between steps.

NTS brainstem slice for electrophysiology

On the day of the electrophysiological experiments, rats with and without previous retrograde tracer application were deeply anesthetized with isoflurane. Horizontal brainstem slices (250 µm) of the NTS that included a sufficient length of the solitary tract to electrically evoke monosynaptic AMPA receptor mediated currents in NTS neurons were prepared as previously described.[9] A patch pipette provided whole cell access to NTS neurons for both current- and voltage-clamp recording protocol. Experiments were carried out at 33-35°C and pH 7.4 with continuous perfusion of an ACSF as previously described.[9] Synaptic currents were evoked through stimulation from a concentric bipolar electrode (FHC, Bowdoinham, ME) placed on the solitary tract. Initial synaptic characterization of NTS neurons was carried out using a burst of five 200 µs current pulses delivered at 50 Hz, with a three second interval between each trail. Latency of ST-evoked EPSCs was calculated as the time from stimulus artifact to the onset of the first EPSC in each train using a differentiated trace.[10] Stimulus intensity was gradually increased until a corresponding train of excitatory post-synaptic currents (EPSC) was recorded in the patched NTS neuron. Tract stimulus intensity was then increased beyond this threshold up to a magnitude 5 times greater. For the duration of the experiment tract stimulus intensity was fixed at between 1.5x to 2.5x threshold. A physiological ACSF with 1 mM $[Ca^{2+}]_o$ ensured that neurotransmission at visceral afferent monosynaptic connections to second-order NTS neurons occurred with a release probability of 70% or less.[11] The readily releasable pool of vesicles would therefore have the capacity for an increase in exocytosis with the elevated Ca^{2+} influx that results from a broadening of the action potential

in the presynaptic terminal.[12]

Recording solutions

For action potential (AP) recordings (isolated neuron): an extracellular solution containing (in mM): 137 NaCl, 5.4 KCl, 1.0 $MgCl_2$, 2.0 $CaCl_2$, 10 glucose, and 10 HEPES with pH adjusted to 7.35 using 1.0 N NaOH. The pipette solution for the recordings contained (in mM): 140 K-aspartate, 3.0 $MgCl_2$, 4.0 BAPTA-K, 10 HEPES, and 0.25 $CaCl_2$ for a final buffered intracellular Ca^{2+} concentration of 100 nM with pH adjusted to 7.3 using 1.0 N KOH. Prior to recording, 2.0 mM Mg-ATP was added to the pipette solution. Osmolarities of extracellular and pipette solutions were adjusted using D-mannitol to 310 and 295 mOsm, respectively. All recordings were performed at room temperature (22-23°C).

For AP and EPSC recordings (NTS brain slice): an artificial CSF (ACSF) composed of the following (in mm): 125 NaCl, 3 KCl, 1.2 KH_2PO_4, 1.2 $MgSO_4$, 25 $NaHCO_3$, 10 dextrose, and 2.0 $CaCl_2$. An electrodes were filled with a solution composed of the following (in mm): 10 NaCl, 110 K-gluconate, 20 KOH, 11 EGTA, 1 $CaCl_2$, 2 $MgCl_2$, 10 HEPES, 1 NaATP, and 0.1 NaGTP, pH 7.3 (295 mOsm). All recordings were performed at near body temperature (35-36°C).

References

1. Qiao GF, Qian Z, Sun HL, Xu WX, Yan ZY, Liu Y, Zhou JY, Zhang HC, Wang LJ, Pan XD, Fu Y. Remodeling of hyperpolarization-activated current, ih, in ah-type visceral ganglion neurons following ovariectomy in adult rats. *PloS one*. 2013;8:e71184
2. Doyle MW, Bailey TW, Jin YH, Appleyard SM, Low MJ, Andresen MC. Strategies for cellular identification in nucleus tractus solitarius slices. *Journal of neuroscience methods*. 2004;137:37-48
3. Li BY, Qiao GF, Feng B, Zhao RB, Lu YJ, Schild JH. Electrophysiological and neuroanatomical evidence of sexual dimorphism in aortic baroreceptor and vagal afferents in rat. *American journal of physiology. Regulatory, integrative and comparative physiology*. 2008;295:R1301-1310
4. Han LM, Ban T, Liu Y, Yuan M, He JL, Wen X, Qian Z, Qiao GF, Li BY. Hyperpolarization-activated current-

mediated slow afterhyperpolarization in myelinated ah-type of baroreceptor neurons isolated from adult female rats. *International journal of cardiology*. 2014;172:e106-108

5. Li BY, Schild JH. Comparisons of somatic action potentials from dispersed and intact rat nodose ganglia using patch-clamp technique. *Acta pharmacologica Sinica*. 2002;23:481-489
6. Li BY, Schild JH. Patch clamp electrophysiology in nodose ganglia of adult rat. *Journal of neuroscience methods*. 2002;115:157-167
7. Li BY, Schild JH. Electrophysiological and pharmacological validation of vagal afferent fiber type of neurons enzymatically isolated from rat nodose ganglia. *Journal of neuroscience methods*. 2007;164:75-85
8. Huo R, Xu WX, Han LM, He JL, Liu Y, Lu XL, Liu SZ, Yuan M, Chen H, Yan JL, Qiao GF, Li BY. Fine tuning of calcium on membrane excitation of baroreceptor neurons in rats. *International journal of cardiology*. 2014;174:883-887
9. Jin YH, Bailey TW, Li BY, Schild JH, Andresen MC. Purinergic and vanilloid receptor activation releases glutamate from separate cranial afferent terminals in nucleus tractus solitarius. *The Journal of neuroscience : the official journal of the Society for Neuroscience*. 2004;24:4709-4717
10. Doyle MW, Andresen MC. Reliability of monosynaptic sensory transmission in brain stem neurons in vitro. *Journal of neurophysiology*. 2001;85:2213-2223
11. Bailey TW, Hermes SM, Andresen MC, Aicher SA. Cranial visceral afferent pathways through the nucleus of the solitary tract to caudal ventrolateral medulla or paraventricular hypothalamus: Target-specific synaptic reliability and convergence patterns. *The Journal of neuroscience : the official journal of the Society for Neuroscience*. 2006;26:11893-11902
12. Schneggenburger R, Meyer AC, Neher E. Released fraction and total size of a pool of immediately available transmitter quanta at a calyx synapse. *Neuron*. 1999;23:399-409
13. Lu XL, Xu WX, Yan ZY, Qian Z, Xu B, Liu Y, Han LM, Gao RC, Li JN, Yuan M, Zhao CB, Qiao GF, Li BY. Subtype identification in acutely dissociated rat nodose ganglion neurons based on morphologic parameters. *International journal of biological sciences*. 2013;9:716-727
14. Doyle MW, Bailey TW, Jin YH, Andresen MC. Vanilloid receptors presynaptically modulate cranial visceral afferent synaptic transmission in nucleus tractus solitarius. *The Journal of neuroscience : the official journal of the Society for Neuroscience*. 2002;22:8222-8229

Supplemental Tables:

Supplemental Table 1: Equal inhibitory effects of 100 ketamine μM on TTX-S and TTX-R components of voltage-gated Na^+ currents functionally expressed on low-threshold and sex-specific subpopulation of myelinated Ah-type baroreceptor neurons housed in nodose ganglia of female rats. The data were read from derivatives; Control: data collected at 1 min after ketamine as control; TTX-S component: the derivative change measured from the baseline to the mid-point of slow portion; TTX-R component: full scale of derivative – TTX-S components. Averaged data were expressed as mean (%) ± SD.

	**1 min (mV/ms)**	**3 min**	**5 min**	**7 min**	**9 min**
**Initial portion**	100% (126.2 ± 27.8)	80.3 ± 9.8	69.8 ± 8.4	49.8 ± 6.7	44.8 ± 6.1
**The portion after**	100% (164.8 ± 33.4)	77.7 ± 8.5	64.9 ± 7.1	52.6 ± 7.4	45.4 ± 5.7

Supplemental Table 2. Effects of ketamine (Ket) on discharge profiles of Ah-type baroreceptor neurons (BRNs) isolated from adult female rats. The Ah-type BRNs (*n* = 9) were isolated from adult female rats and identified electrophysiologically and fluorescently. Both single and repetitive action potential (AP) discharges were evoked by brief pulse and depolarized steps and collected before after Ket that was applied through the bath perfusion (~1 ml/min). Averaged data were presented as mean ± SD. **P* < 0.05 and ***P* < 0.01 vs control.

Parameters	Control	Ket 30 μM	Ket 100 μM	Ket 300 μM
RMP	61.4 ± 3.12	60.8 ± 2.66	60.9 ± 3.04	60.6 ± 2.86
APFT	42.7 ± 4.31	40.4 ± 3.77	36.1 ± 2.17*	34.2 ± 1.97**
AP_{Peak}	58.4 ± 5.27	52.1 ± 4.12*	43.4 ± 3.73**	36.2 ± 3.04**
APD_{50}	1.46 ± 0.56	2.87 ± 0.59**	6.24 ± 1.45**	8.89 ± 2.06**
UV_{MAX}	371 ± 44	292 ± 38*	163 ± 19**	118 ± 14**
DV_{MAX}	82.1 ± 10.4	87.9 ± 9.64	49.1 ± 6.11**	34.6 ± 4.13**
APFF	23.6 ± 6.18	9.86 ± 2.44**	3.68 ± 1.02**	1.25 ± 0.53**

Note: **RMP**: Resting membrane potential (mV). **APFT**: AP firing threshold (mV). **AP_{Peak}**: AP peak (mV). **APD_{50}**: AP duration at 50% deflection (msec). **UV_{MAX}**: the maximal upstroke velocity (mV/ms). **DV_{MAX}**: the maximal downstroke velocity (mV/ms). **APFF**: AP firing frequency (Hz).
